# Neuropsychological Subgroups of Emotion Processing in Youths With Conduct Disorder

**DOI:** 10.3389/fpsyt.2020.585052

**Published:** 2020-12-22

**Authors:** Gregor Kohls, Graeme Fairchild, Anka Bernhard, Anne Martinelli, Areti Smaragdi, Karen Gonzalez-Madruga, Amy Wells, Jack C. Rogers, Ruth Pauli, Helena Oldenhof, Lucres Jansen, Arthur van Rhijn, Linda Kersten, Janine Alfano, Sarah Baumann, Beate Herpertz-Dahlmann, Agnes Vetro, Helen Lazaratou, Amaia Hervas, Aranzazu Fernández-Rivas, Arne Popma, Christina Stadler, Stephane A. De Brito, Christine M. Freitag, Kerstin Konrad

**Affiliations:** ^1^Department of Child and Adolescent Psychiatry, Psychosomatics and Psychotherapy, Child Neuropsychology Section, University Hospital RWTH Aachen, Aachen, Germany; ^2^Department of Psychology, University of Bath, Bath, United Kingdom; ^3^Department of Child and Adolescent Psychiatry, Psychosomatics and Psychotherapy, University Hospital Frankfurt, Frankfurt am Main, Germany; ^4^Centre for Addiction and Mental Health, Toronto, ON, Canada; ^5^King's College London, London, United Kingdom; ^6^School of Psychology, Cardiff University, Cardiff, United Kingdom; ^7^School of Psychology, Institute for Mental Health, University of Birmingham, Birmingham, United Kingdom; ^8^Centre for Human Brain Health, School of Psychology, University of Birmingham, Birmingham, United Kingdom; ^9^Department of Child and Adolescent Psychiatry, Amsterdam Public Health – Mental Health, Vrije Universiteit Amsterdam, Amsterdam, Netherlands; ^10^Psychiatric University Clinics, University of Basel, Basel, Switzerland; ^11^Department of Child and Adolescent Psychiatry, Psychosomatics and Psychotherapy, University Hospital RWTH Aachen, Aachen, Germany; ^12^Child and Adolescent Psychiatry Department, Pediatrics and Child Health Center, University of Szeged, Szeged, Hungary; ^13^Child and Adolescent Unit of the 1st Department of Psychiatry, National and Kapodistrian University of Athens, Athens, Greece; ^14^Child and Adolescent Mental Health Unit, University Hospital Mutua Terrassa, Barcelona, Spain; ^15^Psychiatric Service, Basurto University Hospital, Bilbao, Spain; ^16^JARA-Brain Institute II, Molecular Neuroscience and Neuroimaging, RWTH Aachen and Research Centre Juelich, Juelich, Germany

**Keywords:** conduct disorder (CD), callous-unemotional (CU) traits, limited prosocial emotions specifier, emotion recognition, emotion learning, emotion regulation, neuropsychology, heterogeneity

## Abstract

**Background:** At the group level, youths with conduct disorder (CD) show deficient emotion processing across various tasks compared to typically developing controls (TDC). But little is known about neuropsychological subgroups within the CD population, the clinical correlates of emotion processing deficits [for instance, with regard to the presence or absence of the DSM-5 Limited Prosocial Emotions (LPE) specifier], and associated risk factors.

**Methods:** 542 children and adolescents with CD (317 girls) and 710 TDCs (479 girls), aged 9–18 years, were included from the FemNAT-CD multisite study. All participants completed three neuropsychological tasks assessing emotion recognition, emotion learning, and emotion regulation. We used a self-report measure of callous-unemotional traits to create a proxy for the LPE specifier.

**Results:** Relative to TDCs, youths with CD as a group performed worse in all three emotion domains. But using clinically based cut-off scores, we found poor emotion recognition skills in only 23% of the participants with CD, followed by emotion regulation deficits in 18%, and emotion learning deficits in 13% of the CD group. Critically, the majority of youths with CD (~56%) did not demonstrate any meaningful neuropsychological deficit, and only a very small proportion showed pervasive deficits across all three domains (~1%). Further analyses indicate that established DSM-5 subtypes of CD are not tightly linked to neurocognitive deficits in one particular emotion domain over another (i.e., emotion recognition deficits in CD+LPE vs. emotion regulation deficits in CD–LPE).

**Conclusions:** Findings from this large-scale data set suggest substantial neuropsychological diversity in emotion processing in the CD population and, consequently, only a subgroup of youths with CD are likely to benefit from additional behavioral interventions specifically targeting emotion processing mechanisms.

## Introduction

Conduct disorder (CD) is one of the most prevalent externalizing disorders in childhood and adolescence (1). It is a leading cause of referral to mental health and youth welfare services and incurs enormous healthcare and societal costs (2). Paradoxically, though, CD is one of the least studied, funded, and understood psychiatric disorders in youth (3). Children and adolescents with CD are characterized by severe antisocial and aggressive behaviors that violate age-appropriate societal norms and the rights of others (1). Empirical data emphasize that CD is a highly heterogeneous condition in terms of clinical phenotype (including different subtypes and psychiatric comorbidities), clinical course (i.e., persistent vs. desisting symptomatology), psychosocial outcomes throughout the lifespan, and contributing environmental and dispositional risk factors [see (3) for a comprehensive overview]. Regarding the latter, accumulating evidence suggests that deficits in different emotion processing domains, such as emotion recognition (e.g., difficulties in identifying facial expressions), emotion learning (e.g., difficulties in learning from punishment), and emotion regulation (e.g., difficulties in inhibiting impulsive responses to emotional cues), may offer a particularly powerful basis for explaining potentially different presentations and trajectories of CD behaviors, including aggression (4–8). For instance, deficits in the recognition of distress cues, such as emotional expressions of fear, sadness or pain, but also of other facial expressions, such as happiness, appear to be most pronounced in individuals with CD who have high levels of callous-unemotional (CU) traits (i.e., reduced guilt and empathy, callousness, and uncaring attitudes) (9). Individuals with CD who present with at least two of these CU traits fulfill criteria for the Limited Prosocial Emotions (LPE) specifier in DSM-5 (1). This subtype of CD is considered particularly severe as affected individuals typically present with an earlier age-of-onset and a more serious and stable set of symptoms, including proactive aggression, placing them at increased risk for poor treatment outcomes (10) [but see also (11, 12)] and for developing mental health problems in adulthood, such as antisocial personality disorder (ASPD) (6, 13, 14). In contrast, individuals with CD but without the LPE specifier (i.e., those showing subclinical levels of CU traits) are thought to show emotion regulation deficits, such as an inability to maintain behavioral control when confronted with acute emotional stimuli (e.g., visual threats), which may contribute to impulsive acts of reactive aggression and an increased risk for anxiety and depression (6). Finally, emotional learning deficits, such as a failure to learn how to avoid choices that lead to punishment rather than reward, occur more broadly in youths with CD irrespective of their LPE status (15).

However, most prior work on emotion functioning in CD, and its clinically defined subtypes, has been limited by relying on relatively small samples with varying selection criteria and neurocognitive tasks (16), including mixed samples of youths with CD or oppositional defiant disorder (ODD), or focusing on a single subdomain of emotion dysfunction instead of all three domains linked to CD, including emotion recognition, learning, and regulation (4, 5, 7). Thus, studies to date have largely been unsuited or underpowered for testing within-CD, individual variability of the underlying neurocognitive disease mechanism(s), including emotion dysfunction. To address the above-mentioned research gaps, we initiated the largest study to date to comprehensively investigate emotion recognition, emotion learning, and emotion regulation using a broad neuropsychological test battery within a single sample of youths with CD (*n* = 542) compared to typical controls (*n* = 710) (17). As traditionally done in this line of research, we first compared the group of youths diagnosed with CD with the typical controls, and based on statistically significant group differences or the lack thereof, we determined whether a CD-related neurocognitive deficit was present or not. As expected, we found that emotion deficits in the CD group spanned across the three neurocognitive domains. However, we also noted that the significant group differences between CD and controls in task performance had effect sizes in the small to very small range (i.e., Cohen's *d*s <0.29). As this is in line with previous meta-analytic findings (18, 19), these results indeed suggest: (i) substantial distributional overlap *between* the CD and non-CD samples in terms of performance on emotion processing tasks; and (ii) substantial variation in emotion processing abilities *within* the CD population. Hence, it is reasonable to assume that the significant CD-vs.-control effects for emotion processing tasks—or any other neuropsychological measure reported in the literature [e.g., (20)], may either be truly small effects driven by the entire CD sample or, which appears more likely, they are driven by only a subset of youths with CD who have emotion processing deficits (21).

In fact, this notion of diversity in emotion processing is emphasized by current neurocognitive models of CD etiology [e.g., (6, 7)]. These models suggest that dysfunction in distinct emotion processing domains are associated with different subtypes of CD and related symptom sets (22). For instance, given the assumption that youths with CD with the LPE specifier show difficulties in perceiving other people's emotions, particularly distress and happiness, one might predict that performance in this neurocognitive domain would be disproportionately deficient in this subgroup, whereas the subgroup of youths with CD but without the LPE specifier would show specific difficulties with emotion regulation (4, 6).

In addition, emotion dysfunction might serve as an “intermediate phenotype”—i.e., developmental neurocognitive mechanism (23)—linking risk for psychopathology with the emergence of clinical symptomatology, including clinical subtypes of CD (24). There are multiple dispositional and contextual risk factors that have repeatedly been implicated in CD, such as birth complications, maladaptive parenting, or low socioeconomic status [reviewed in (3)]. Data from epidemiological and at-risk samples suggests that particular risk factors appear to have closer associations with a specific domain of emotion dysfunction (25). For instance, children exposed to physical violence or abuse exhibit altered emotion recognition processes (26), including an altered ability to identify and discriminate specific emotions (i.e., anger) contributing to ‘hostile attribution biases' (i.e., misinterpreting neutral or ambiguous facial expressions as threatening), which, in turn, predict the emergence of CD behaviors, such as aggression (27, 28). In slight contrast, exposure to numerous adversities, such as poverty (incl. low socioeconomic status), deprivation (incl. institutional rearing), maltreatment, or pre- and perinatal influences (incl. maternal smoking during pregnancy, or birth complications), appears to be related to emotion regulation and learning difficulties predicting the onset of both externalizing and internalizing problems (29). Although the literature is far from being conclusive in linking adversity factors with specific neurocognitive processes as intermediate phenotypes of conduct problems, studying neuropsychologically defined subtypes of emotion dysfunction in CD may provide novel insights into mechanisms that presumably underlie the complex developmental pathways from risk for psychopathology to different clinical expressions of the disorder (30).

Thus, the primary aim of the current study was to adopt a clinically motivated, person-centered (rather than variable-centered) bottom-up analytic approach to explore the neurocognitive diversity of emotion functioning in CD. We accomplished this by re-analyzing the neuropsychological task performance data from our large sample of girls and boys with CD who were comprehensively clinically assessed and reliably diagnosed using standardized, semi-structured interviews (17, 31). For each of the three emotion processing tasks that assessed emotion recognition, emotion learning, and emotion regulation, respectively, we defined deficit as task performance within the bottom 10% of an age-matched control group (equivalent to approximately 1.3 standard deviations below the mean), following the common-metric approach usually applied in pediatric neuropsychology and as previously used in neurocognitive studies in ADHD (21, 32–35).

To our knowledge, this study is the first to investigate neuropsychological subgroups within the CD population by exploring the proportion of youths with CD who do vs. those who do not have deficits in emotion processing, including emotion recognition, emotion learning, and emotion regulation (but see (36, 37) for similar approaches focusing on other neurocognitive domains, such as verbal skills, mental flexibility, or memory, in smaller-scale studies). Our sample is particularly suited to investigate neurocognitive diversity within CD as it is one of the largest, most representative and clinically well-characterized cohorts of girls and boys with CD (vs. typical controls) recruited from a variety of sources, including the community, specialist schools, mental health clinics, welfare institutions, and youth offending services in different European countries (31). Given the magnitude of the effect sizes observed in our previous study (17), we expected to find subgroups of youths with CD without deficit in any domain vs. those who have deficits in only one domain, two domains, or across all three domains. Most importantly, we tested the extent to which neuropsychologically defined subgroups would map clinically onto the CD subtypes described in the DSM-5, including CD with vs. without the LPE specifier, and as a secondary aim, we explored whether the neuropsychological subgroups would be associated with specific CD-related risk factors.

## Materials and Methods

### Participants

As part of the European multi-site project entitled “Neurobiology and Treatment of Adolescent Female Conduct Disorder: The Central Role of Emotion Processing” (FemNAT-CD; https://cordis.europa.eu/project/id/602407/reporting), we reanalyzed the neuropsychological data obtained from our large sample of youths with CD (*n* = 542, 317 girls) and TDCs (*n* = 710, 479 girls), aged 9–18 years (see (17) for details on recruitment, clinical assessments, and sample characteristics). In brief, we used data from participants who provided a complete neuropsychological dataset which included facial emotion recognition (*Emotion Hexagon task*), emotion learning (*Passive Avoidance Learning task*), and emotion regulation skills (*Emotional Go/Nogo task*). Participants were recruited through community outreach (e.g., mainstream schools) as well as from mental health clinics, welfare institutions, and youth offending services at 10 sites across Europe (Supplementary Table 1) (31). Overall exclusion criteria were IQ <70, autism spectrum disorders, schizophrenia, bipolar disorder or mania, neurological disorders, and genetic syndromes. Individuals with CD were diagnosed according to DSM-IV-TR criteria (38). Youths with “only” ODD who did not fulfill the diagnostic criteria for CD were excluded from the current analysis. TDCs were free of current psychiatric diagnoses and lifetime diagnoses of CD, ODD, and ADHD. We excluded TDCs with lifetime histories of and/or current disruptive behavior disorders, such as ADHD, ODD, and CD, in order to rule out the influence of any subclinical or precursor symptoms that are potentially linked to CD. Written informed consent was obtained for all participants, and local ethics committees approved the study protocol. Table 1 summarizes the sample's main demographic and clinical characteristics.

**Table 1:** Demographic and clinical characteristics.

	**CD *n*=542**	**TDC *n*=710**	**Group effect *p-values^a^***
**Age (years)** ***M*** **(SD)**	14.4 (2.3)	14.0 (2.5)	0.001
**Females (%)**	58.5	67.5	0.001
**Estimated IQ** ***M*** **(SD)**	94.9 (12.4)	103.5 (12.2)	<0.001
**SES** ***M*** **(SD)**	−0.29 (0.93)	0.28 (1.03)	<0.001
**CD total symptoms** ***M*** **(SD)**	5.45 (2.34)	0.05 (0.23)	<0.001
**Average age-of-onset of CD (years)** ***M*** **(SD)**	10.3 (3.8)	N/A	
**CD age-of-onset subtype (%)**			
Childhood	43.0	N/A	
Adolescence	53.3	N/A	
Unspecified	3.7	N/A	
**CD severity (%)**			
Mild	24.3	N/A	
Moderate	52.8	N/A	
Severe	20.5	N/A	
Unknown	2.4	N/A	
**Impairment caused by current CD (%)**			
With peers	63.7	N/A	
With family	85.9	N/A	
With school	78.9	N/A	
Unknown	1.2	N/A	
**LPE specifier (%)**	43.7	18.3	<0.001
**Current comorbidities** ***n*** **(%)**			
ODD	78.2	N/A	
ADHD	38.4	N/A	
SUD	17.4	N/A	
MDD	14.8	N/A	
PTSD	6.7	N/A	
GAD	3.0	N/A	
**Psychotropic meds (%)**	30.2	N/A	

*Diagnoses and CD symptoms were based on the Schedule for Affective Disorders and Schizophrenia for School-Age Children–Present and Lifetime version (K-SADS-PL). For TDC, any current psychiatric diagnosis as well as a history of ADHD, ODD, or CD was exclusionary. ADHD, attention deficit hyperactivity disorder; CD, conduct disorder; GAD, generalized anxiety disorder; IQ, estimated intelligence quotient; LPE, limited prosocial emotions specifier [see (39)]; MDD, major depressive disorder; Meds, on psychotropic medications; ODD, oppositional defiant disorder; PTSD, post-traumatic stress disorder; SES, socioeconomic status (SES was based on parental income, education level, and occupation (40)); SUD, substance use disorder (including substance abuse and dependence); TDC, typically developing controls*.

a *p-values are based on two-sample t-tests or χ*^2^* tests*.

All individuals were clinically assessed with the Kiddie-Schedule for Affective Disorders and Schizophrenia–Present and Lifetime version [K-SADS-PL (41)]. The K-SADS-PL is a semi-structured clinical interview that is administered separately to caregivers and participants by trained staff members to assess current and lifetime psychiatric diagnoses, disorder severity, and age-of-onset and duration of a disorder. Additionally, where available, information from medical or case files was used. Summary ratings were derived from the clinical judgment using all sources. The items of the K-SADS-PL are scored on a scale from 0 to 3. A rating of 0 indicates no (insufficient) information, a score of 1 indicates a given symptom is not present, 2 indicates a subclinical expression, while a score of 3 is given when a symptom is present and clinically significant. Scores were recoded, so that a clinical rating of “not present” is represented by 0, a subclinical rating by a score of 1, and a clinically significant rating by a score of 2. Inter-rater reliability (IRR; *N* = 75, i.e., *n* = 5–8 per site) of CD was high (Cohen's κ = 0.91), with an agreement rate of 94.7%. IRR of other disorders, including ADHD, ODD, major depressive disorder (MDD), and generalized anxiety disorder (GAD), was also high (Cohen's κs ≥ 0.84, agreement rates ≥92%), which is in line with the reliability data reported by Kaufman et al. (41). These authors also report data which support the concurrent validity of the diagnoses generated with this instrument. Youths who met criteria for a specific disorder (e.g., behavior disorder) scored significantly higher than undiagnosed youths on rating scales assessing related symptom sets. Using the K-SADS-PL, we also determined the CD-onset type [i.e., childhood-onset (CO-CD): presence of at least one CD symptom and impairment prior to age 10; adolescent-onset (AO-CD): CD symptoms only emerge after age 10] (1).

Full-scale IQs were estimated using the vocabulary and matrix reasoning subtests of the Wechsler Intelligence Scale for Children-Fourth Edition (42), the Wechsler Adult Intelligence Scale-Fourth Edition (43), or the Wechsler Abbreviated Scale of Intelligence (44). The vocabulary subtest consists of 31 items, and youths are required to verbally define and/or describe a word or concept that is orally presented to them. Each item is scored on a 0-, 1-, or 2-point basis according to the manual. In the matrix reasoning subtest 30 visually-depicted incomplete matrices are presented, and youths are required choose one item from a selection of five options that correctly completes the matrix. Each correct item receives 1 point. The *T* and standard scores for each subtest were transformed into *z*-scores and then combined to yield estimates of full-scale IQ. For the two-subtest short form (FSIQ-2) internal consistency and test-retest reliability were reported to be excellent (>0.90). Estimated full-scale IQ scores were highly correlated with scores on tests purported to measure similar constructs. Correlations between the short form and the original tests were reported to be acceptable (0.71) to excellent (0.92) (45).

CU traits scores were derived from the Youth Psychopathic traits Inventory (YPI) (46). The YPI is a 50-item self-report measure of psychopathic traits. Each item is answered on a 4-point Likert scale ranging from “does not apply at all” (1) to “applies very well” (4). Higher scores indicate higher levels of psychopathy. CU traits scores were calculated using the total score for the subscales ‘remorselessness' (e.g., “*To feel guilt and regret when you have done something wrong is a waste of time”*), ‘unemotionality' (e.g., “*I usually feel calm when other people are scared”*), and ‘callousness' (e.g., “*I think that crying is a sign of weakness, even if no one sees you”*). The CU traits dimension showed good internal consistency (Cronbach's α = 0.81). Test-retest reliability of the YPI over a 6-month period was reported to be adequate (*ICC* = 0.76) (47). Convergent and divergent validity was supported in a sample of 360 youths from the general population (e.g., CU traits scores correlated positively with narcissism, but negatively with empathy measures). We also used the three CU traits subscales of the YPI to create a proxy for the LPE specifier, following the procedure developed by Colins and Vermeiren (39). A participant was considered to meet criteria for one of the CU traits when she/he reported that at least one item on the corresponding subscale applied “very well” to her/him [i.e., a score of 4 on a 4-point Likert scale, ranging from “Does not apply at all” (1) to “Applies very well” (4)]. Participants were considered to meet criteria for the LPE specifier if two or more CU traits were endorsed to threshold.

Participants reported on their own aggressive behaviors using the Reactive-Proactive aggression Questionnaire (RPQ) (48), which includes 11 items related to ‘reactive aggression' (e.g., “*I have damaged things because I felt mad”*), and 12 items related to ‘proactive aggression' (e.g., “*I have had fights to show that I was on top”*). Each item is rated on a 3-point Likert scale ranging from 0 (“never”) to 2 (“often”). The proactive and reactive aggression scales are sum scores of the respective items. Internal consistency for the two subscales was good (Cronbach's α = 0.75 and 0.88, respectively). Raine et al. (48) also report data which support the validity of the two subscales.

In addition to gender/sex (i.e., male) and general cognitive abilities (i.e., low IQ), numerous longitudinal studies (e.g., (49, 50)) have identified several risk factors linked to CD (reviewed in (3)) of which the following were assessed in the present study: maternal smoking during pregnancy, parental maladaptive behavior (i.e., repeated delinquency of mother/father), and socioeconomic status [i.e., SES (40)]. These three variables were extracted from the Medical History Questionnaire which is a semi-structured interview for parents/caregivers specifically designed for this study with items included based on evidence about CD-related risk factors derived from epidemiological studies (31). Additionally, childhood exposure to parental violence/abuse/neglect, and deviant peer affiliations were evaluated with the Childhood Experience of Care and Abuse Questionnaire (CECA-Q, i.e., total sum of the subscale scores for “antipathy mother/father,” “neglect mother/father,” and “physical abuse mother/father”, Cronbach's α = 0.78) (51), and the Social and Health Assessment (SAHA, i.e., “affiliation with delinquent peers” subscale) (52), respectively. The CECA-Q is a self-report questionnaire to assess lack of parental care (neglect and antipathy), parental physical abuse, and sexual abuse from any adult (not used in this study) before age 17. Satisfactory reliability and validity have been reported by Bifulco et al. (51). The SAHA subscale consists of nine items, and youths are asked about how many of their close friends are involved in different types of risk-taking and delinquent behavior (“None”; “A few”; “Some”; or “Most or all”): e.g., dropping out of school, smoking cigarettes, drinking alcohol, or using marijuana. The summed score could range from 9 to 36 where higher scores indicate greater association with delinquent peers. The internal consistency was high (Cronbach's α = 0.91), and published data support the validity of this subscale (53).

### Neuropsychological Test Battery

We used the *Emotion Hexagon task* to assess the accuracy of facial emotion recognition (54). Participants were asked to label morphed facial expressions as either happy, sad, angry, fearful, disgusted, or surprised (i.e., the six “basic” emotions). Morphs were created from six expression pairings: happy-surprised, surprised-fearful, fearful-sad, sad-disgusted, disgusted-angry, and angry-happy. Each pair included two prototype expressions in proportions 90:10, 70:30, 50:50, 30:70, and 10:90 (i.e., 10% happy and 90% surprised for the happy-surprised continuum). Morphed expressions were presented individually and randomly on a computer monitor for a maximum of 3 s, and participants were asked to select by mouse-click one of the six emotion labels that best described the expression shown. Participants were given as long as necessary to make their selection and were not given feedback about their performance accuracy. Participants completed one practice block, followed by five blocks that each displayed all 30 morphed expressions once (6 pairs x 5 morphs). The total score for incorrect recognition per expression ranged from 20 (100% error rate) to 0 (0% error rate), with 50:50 morphs not being scored or analyzed.

We administered a modified *Passive Avoidance Learning task* to assess the accuracy of emotional learning (55). The task involves assigning reward and punishment values to novel stimuli (“ziggerins” (56)). Novel stimuli were chosen to tap into pure learning effects without the bias of stimulus familiarity. Participants were instructed to learn by trial-and-error to respond through button press to four different reward stimuli (gaining 1, 700, 1,400, or 2,000 points, respectively; non-responses were counted as omission errors in %) and to avoid responding to four different punishment stimuli (losing 1, 700, 1,400, or 2,000 points, respectively; responses to these stimuli were counted as avoidance errors in %). Each stimulus was shown once within a block of 8 trials, with 10 blocks overall (including one practice block). Stimuli were displayed on a computer monitor for a maximum of 3 s, followed by performance feedback (i.e., amount of points won, or lost, as well as the running total points). Participants started the task with 10,000 points.

We administered the *Emotional Go/Nogo task* to assess the accuracy of emotion regulation defined as the ability to maintain cognitive control when confronted with interfering emotional information, including positive and negative facial expressions (57, 58). Participants were instructed to press a response button as quickly and accurately as possible whenever a named facial expression appeared on the screen (go trials) and not to press for any other expression (nogo trials). The task included six randomly presented blocks of go-nogo pairings: neutral-happy, neutral-fearful, happy-neutral, fearful-neutral, happy-fearful, and fearful-happy. Each block included 35 go (73%) and 13 nogo (27%) stimuli. The go trials occurred more frequently in order to create a pre-potent tendency for the participant to respond. Stimuli consisted of gray-scaled fearful, happy, and neutral expressions from six male and six female adults, with four African-American, Asian, and Caucasian individuals for each expression type, respectively (NimStim set numbers: 6, 8, 11, 14, 15, 16, 27, 36, 39, 43, 44, and 45). Stimulus duration was 500 ms with 1 s interstimulus intervals. False alarm error rates in % for nogo trials indexed emotion regulation, with higher rates reflecting worse performance (59).

**Figure 1:**
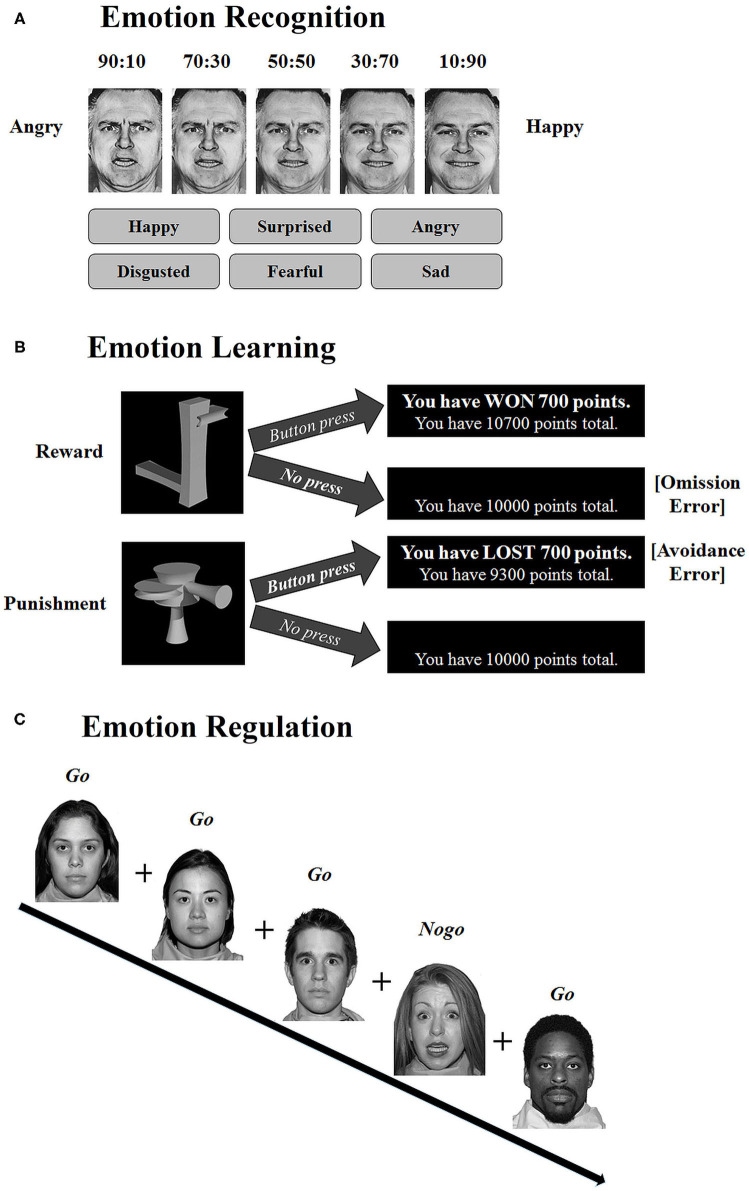
Illustration of the model based neuropsychological test battery used to assess **(A)** emotion recognition, **(B)** emotion learning, and **(C)** emotion regulation, respectively. **(A)** As an example, the angry-happy facial expression continuum from the *Emotion Hexagon task* is depicted, including the five different morphs from this continuum as well as the six emotion labels used in the task. Only one facial expression is displayed in each trial. **(B)** Examples from the *Passive Avoidance Learning task*, depicting one stimulus associated with reward (e.g., gaining 700 points by button press), and one stimulus associated with punishment (e.g., losing 700 points by button press). **(C)** Example layout of the emotion regulation condition from the *Emotional Go/Nogo task*, including neutral expressions as the “Go” targets and fearful expressions as the “Nogo” non-targets. This Figure was republished from Kohls et al. (17) with permission from ELSEVIER.

Order of tasks was pseudorandomized separately across group (CD, TDC), sex (female, male), and age brackets (9–12, 13–15, and 16–18 years). The extracted performance variables of the three tasks (see Table 1) had acceptable to good reliabilities (Cronbach's α ≥ 0.70). Details on the test battery and procedures are provided in Figure 1 [for more details, see also (17)]. We chose this particular test battery based on influential models of emotion dysfunction in CD (see (17) for details), and because the three tasks have widely been used in neuropsychological research of emotion functioning in developmental psychopathology, including CD, ADHD, and internalizing disorders. Thus, the validity of the test battery comes from its proven usefulness to distinguish between clinical groups and controls in previous research (4). Available psychometric data further support both reliability and validity of all three neuropsychological measures (59–61). Standard operating procedures (SOP) ensured consistency of data collection, handling, and analysis across all data collecting sites.

### Statistical Analyses

First, all raw scores for the performance variables of interest from our neuropsychological battery (see above, and Table 2 below) were age-, IQ-, and sex-adjusted using standard regression procedures, resulting in *z*-scores as the dependent variables in the following factor analysis (conducted in SPSS v25, IBM Corp., Armonk, NY). We ran a confirmatory principal component (PCA) “factor” analysis, using varimax rotation with Kaiser normalization, as we had *a priori* expectations about the number of factors that would be associated with the measured dependent variables (62), i.e., separate factors for emotion recognition, emotion learning, and emotion regulation, respectively. All participants were included in the PCA in order to maximize statistical power as well as to create a common metric of performance scores by which youths with CD and TDCs could be compared. Per component, factor scores were extracted for each participant using the Anderson-Rubin method (M = 0, SD = 1) to avoid multicollinearity. We then explored case-control differences for the three emotion domains within a repeated-measures analysis of variance model using the factor scores as the dependent variables. Effect sizes were calculated using partial eta squared (*η*^2^_p_), where 0.01, 0.06, and 0.14 represent small, medium and large effects, respectively (63). We additionally report 95% confidence intervals (CIs) for all effect size measures. Finally, and most importantly, in keeping with the clinically motivated subgrouping approach that has been very informative in the ADHD field (21, 32–35), we subdivided the individuals with CD into subgroups on the basis of their neuropsychological task performance. For each emotion processing domain identified in the factor analysis, participants were classified as “deficient” or “intact,” with deficit being defined as performance (i.e., factor scores) within the bottom 10% of an age-matched control group using the following age brackets to recognize that test performance typically improves with age (64): 9–12, 13–15, and 16–18 years. We then investigated the proportion of youths with CD who had one or multiple deficits vs. no deficit at all. We had three reasons for choosing the 10th percentile criterion as a reasonable cutoff for deficit: First, we wanted to use a clinically useful definition of performance deficit that is established in the field of clinically oriented neuropsychological assessments as this is the core interest of person-centered approaches; second, to apply a threshold which is easily reproducible and comparable between studies within and across disorders (in contrast, for instance, to more elaborate analytic approaches based on machine learning algorithms which usually require specialized expertise that is not available in all research labs or clinics (65)); and third, any other reasonable cutoff (e.g., 5th percentile) would still classify many youths with CD as “intact” or conversely classify a substantial number of typically developing controls (TDC) as “deficient” (i.e., the positive predictive value increases, but the negative predictive value decreases with more stringent cutoffs) (21). Regression analyses, chi-square tests, and *t*-tests were then used to explore differences in clinical correlates and risk factors between the neuropsychologically defined CD subgroups.

**Table 2:** Results from the confirmatory principal component analysis.

	**Rotated “factor” loadings**		
**Variables**	**Emotion regulation**	**Emotion learning**	**Emotion recognition**
**False alarm error rate in % (Go/Gogo pairings)**			
Neutral/Happy	0.783		
Neutral/Fearful	0.764		
Fearful/Happy	0.746		
Happy/Neutral	0.744		
Fearful/Neutral	0.726		
Happy/Fearful	0.723		
**Error rate in % (Punishment, and Reward conditions)**			
Losing 1,400 points		0.724	
Losing 700 points		0.712	
Losing 1 point		0.703	
Losing 2,000 points		0.670	
Gaining 1 point		−0.686	
Gaining 1,400 points		−0.659	
Gaining 700 points		−0.535	
Gaining 2,000 points		−0.476	
**Error rate in % (Emotion expression)**			
Happiness			0.718
Surprise			0.704
Fear			0.667
Sadness			0.632
Disgust			0.605
Anger			0.590
**Eigenvalues**	**3.48**	**3.44**	**2.71**

## Results

### Correlational and Principal Component Analyses

As expected, correlations between the dependent variables (i.e., age-, IQ-, and sex-adjusted performance scores) were larger within each of the three emotion domains (mean *r*_Olkin &Pratt_ = 0.37, 95% CI: 0.34, 0.44) than between the domains (mean *r*_Olkin&Pratt_ = 0.12, 95% CI: 0.07, 0.17; Fisher's *z* = 7.28, *p* <0.001), indicating that our test battery did indeed capture emotion processing as a multifaceted construct rather than a unitary one. For the PCA, the Kaiser-Meyer-Olkin (*KMO*) measure confirmed that the sample size was adequate for the analysis [*KMO* = 0.86, which is a sufficiently high value (66)], and the *KMO* values for all dependent variables were substantially higher than 0.5 (i.e., *KMO*s ≥ 0.68). Bartlett's Test of Sphericity indicated that correlations between variables were large enough for PCA [χ*^2^* (190) = 7514.62, *p* <0.001]. The dependent variables from the three tasks loaded onto the predicted three components, accounting for 48.1% of the total explained variance in performance scores. Components 1, 2, and 3 represented emotion regulation (17.4% variance), emotion learning (17.2% variance), and emotion recognition (13.5% variance), respectively (Table 1). Note: Using other factor analytic procedures, such as the Maximum Likelihood method or Principal Axis Factoring, yielded similar results which is in line with prior work (67) (data available on request).

### Comparing Dimensionally the CD and TDC Groups in Factor Scores

**Figure 2:**
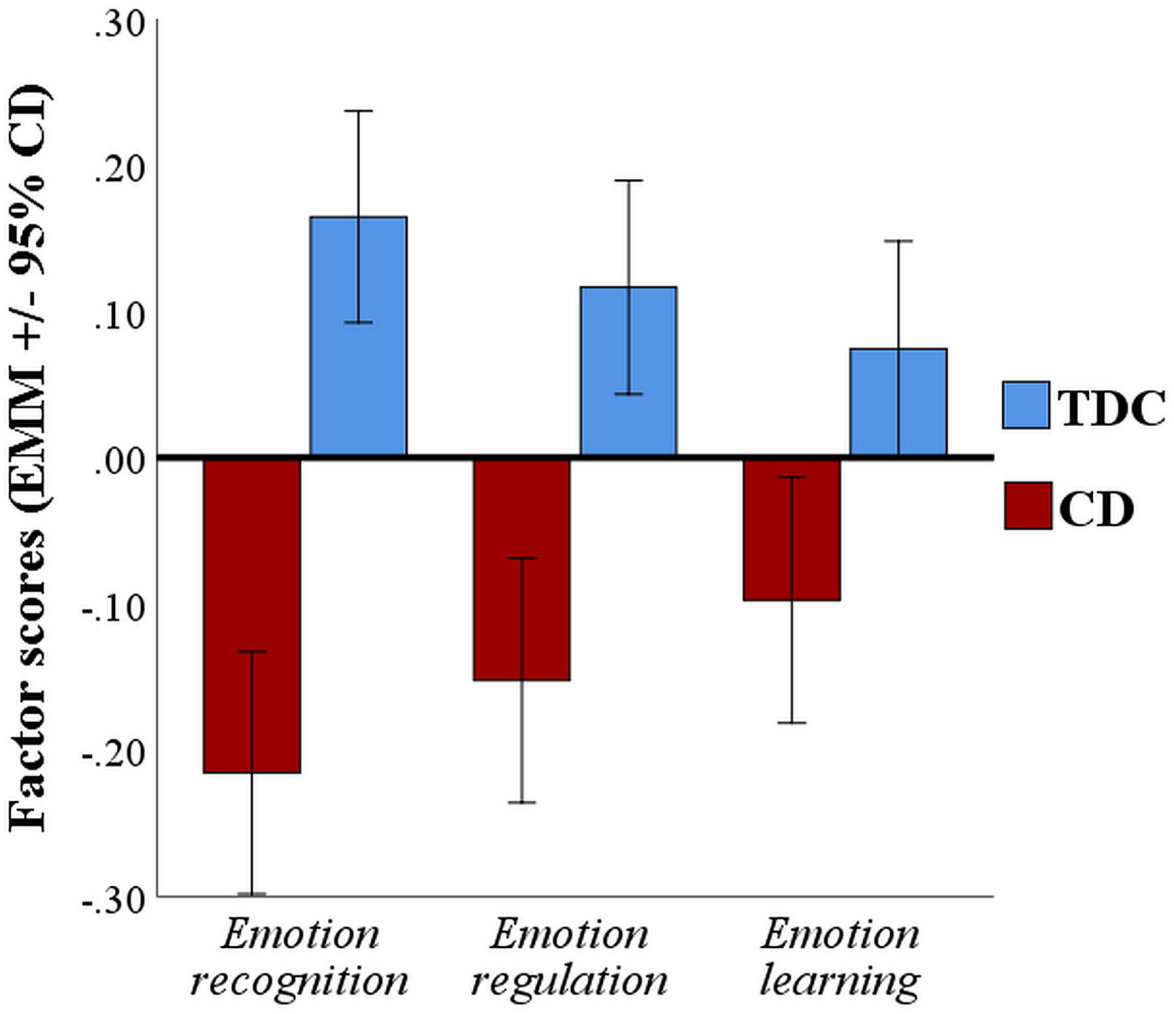
Factor scores for youths with CD vs. TDCs for the three emotion processing domains investigated in this study. Relative to TDCs, youths with CD showed the expected deficits in all three domains, with the greatest deficits in the emotion recognition domain, followed by the emotion regulation and emotion learning domains (EMM, Estimated Marginal Means; 95% CI = error bars represent 95% Confidence Intervals).

We analyzed the factor scores for the three emotion processing domains using a three (domain: emotion regulation vs. emotion learning vs. emotion recognition) by two (group: CD vs. TDC) repeated-measures analysis of variance model, followed by *post-hoc* pairwise comparisons in cases where significant main or interaction effects emerged (Bonferroni-corrected for multiple comparisons). This analysis revealed a small but significant group by domain interaction effect [*F*_(2,500)_ = 3.34, *p* = 0.036, *η*^2^_p_ = 0.003, 95% CI: 0.0001, 0.008], and a significant main effect of group with a medium effect size [*F*_(1,250)_ = 72.98, *p* <0.001, *η*^2^_p_= 0.055, 95% CI: 0.033, 0.081]. Compared to TDCs, youths with CD as a group performed worse in all emotion domains (in line with our prior analyses examining task performance separately for each neuropsychological task (17)), but the largest case-control differences were found for the emotion recognition domain (*η*^2^_p_= 0.035, 95% CI: 0.018, 0.058), followed by the emotion regulation domain (*η*^2^_p_= 0.018, 95% CI: 0.006, 0.035), with the smallest difference observed for the emotion learning domain (*η*^2^_p_= 0.007, 95% CI: 0.0009, 0.019) (Figure 2). We also explored the extent to which group membership (i.e., CD or TDC) was predicted by the three emotion domain scores using a logistic regression analysis. This analysis revealed significant effects of all three emotion domains (*Wald* χ*^2^*s > 9.43, *p*s ≤ 0.002). However, the final model [χ*^2^* (3) = 77.78, *p* <0.001; Hosmer-Lemeshow-Test: *ns*] successfully predicted group membership of 83% (95% CI: 79.8, 85.5%) of the TDCs, i.e., relatively high specificity, but only 36% (95% CI: 31.8, 40.0%) of the CD cases, i.e., relatively low sensitivity, again pointing to substantial variability in emotion processing skills within the CD group.

### Neuropsychologically Based Subgroup Analysis

#### Proportion of Deficit

**Figure 3:**
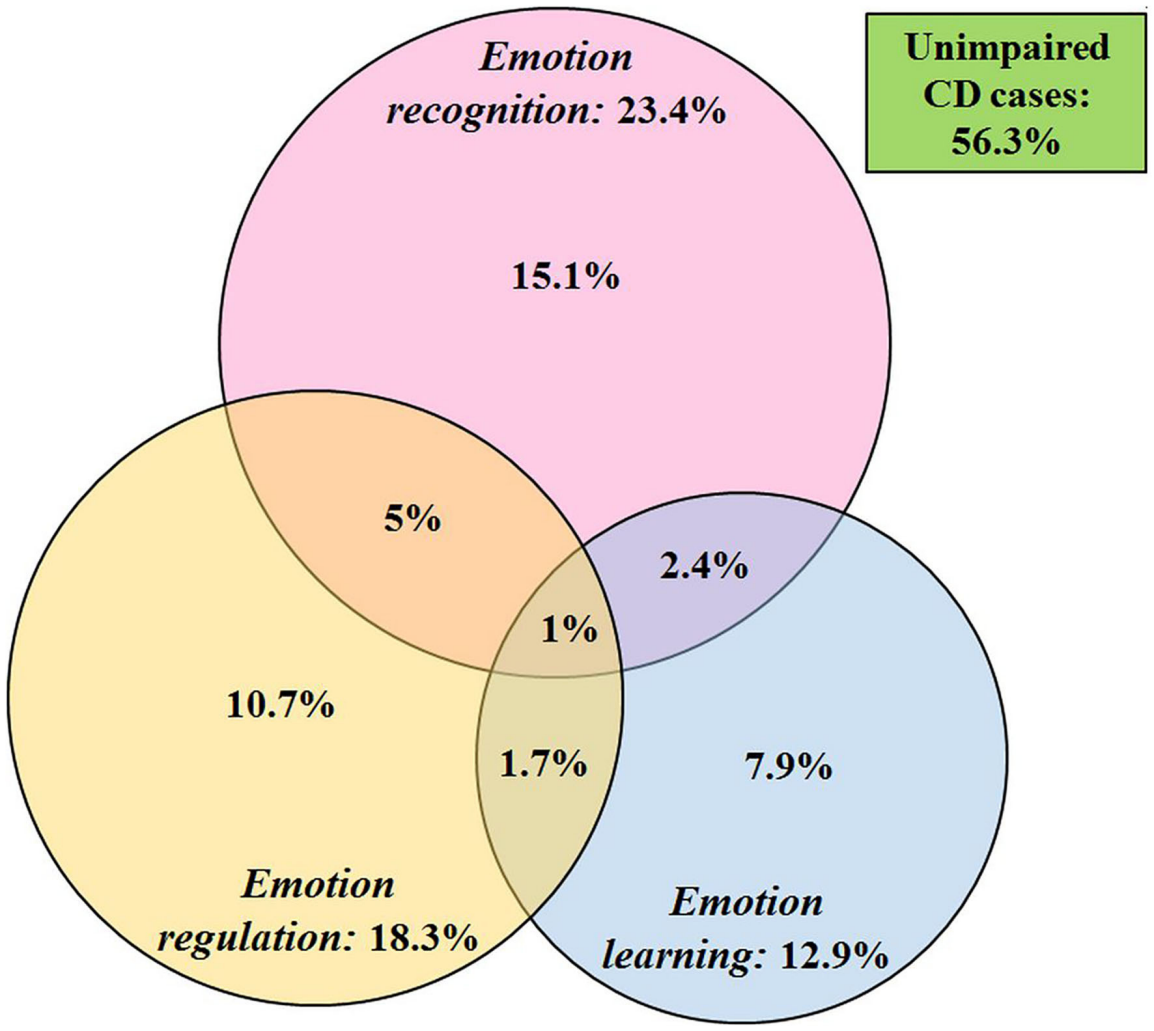
Proportion of CD cases (*n* = 542) with deficits in emotion recognition, regulation, and learning, and their degree of overlap. The numbers reported next to the domain labels reflect the sum of those with a deficit in that particular domain, including those with single, or multiple deficit(s).

Figure 3 presents a Venn diagram showing the proportion of CD cases who exceeded the threshold for one or multiple deficits in the emotion recognition, emotion learning, and/or emotion regulation domains, with deficit being defined as performance (i.e., factor scores) within the bottom 10% of their respective age-matched control group (which equals ≤ -1.3 standard deviations from the mean of performance data by TDCs). In the CD group, 43.7% of the participants were deficient in at least one domain of emotion processing. Deficits in emotion recognition were the most common deficit, followed by emotion regulation, and emotion learning was the least common deficit. Overlap between the different deficits was rare, with only ~1% of the youths with CD displaying a pervasive deficit across all three domains. Compared to TDCs, “deficit” was significantly more frequent among the youths with CD for the emotion recognition and emotion regulation domains (*p*s <0.001), but not for the emotion learning domain (*p* = 0.13). Notably, a substantial subgroup of CD youths (56.3%) showed no deficit in their emotion processing abilities across the three domains. Among the TDCs, ~27% showed at least one emotion deficit (i.e., 24.6% qualified for only one, 2.4% for two, and 0.1% for three deficits), leaving ~73% of the TDCs who performed normally across the three emotion domains (Note: If the three neuropsychological tasks were completely independent, one would expect to find ≥30% of TDCs to be deficient in at least one emotion domain given the 10th percentile criterion as our cutoff for deficit per task).

#### Clinical Correlates

Introducing the three emotion deficit domains (i.e., deficit: yes = “1,” or no = “0”) as predictors into multiple linear or logistic regression models—i.e., running one model with the three predictors for each dependent variable separately—did not reveal any significant associations with the main clinical variables of interest among the youths with CD [i.e., CD symptom severity, CD age-of-onset subtype, CU traits/LPE specifier, or the presence of major comorbidities, including ADHD, ODD, major depressive disorder (MDD), generalized anxiety disorder (GAD), post-traumatic stress disorder (PTSD), and substance use disorder (SUD)]. More specifically, the subgroup of youths with CD with an emotion recognition deficit did not differ significantly from those without such deficit regarding: (i) the presence of the LPE specifier (44.1% vs. 43.6%, *p* = 0.92), (ii) CD age-of-onset subtype (CO-CD/AO-CD: 43.3%/52.8% vs. 42.9%/53.5%, *p* = 0.98), (iii) number of CD symptoms (K-SADS-PL CD symptom count: 5.6 ± 2.4 vs. 5.4 ± 2.3, *p* = 0.57); or (iv) proactive aggression (RPQ proactive aggression subscale score: 5.0 ± 5.2 vs. 4.7 ± 4.6, *p* = 0.56). Moreover, the subgroup of youths with CD with an emotion regulation deficit did not differ significantly from those without such deficit in terms of: (i) the LPE specifier (38.8% vs. 44.8%, *p* = 0.31), (ii) the presence of comorbid anxiety disorders (16.2% vs. 11.7%, *p* = 0.24), or (iii) scores on the RPQ reactive aggression subscale (12.0 ± 4.7 vs. 12.1 ± 5.0, *p* = 0.86). Taken together, these findings suggest that deficits in emotion processing related to CD (4) do not map neatly onto established DSM-5 subtypes, such as CD+LPE or childhood-onset CD.

#### Associated Risk Factors

As a secondary aim, we explored associations between the three emotion processing domains and established risk factors for CD within three logistic regression models, i.e., one for each neurocognitive domain (deficit: yes = “1,” or no = “0”) using the following predictors: gender/sex, general cognitive abilities (i.e., low IQ), maternal smoking during pregnancy, socioeconomic status (i.e., low SES), parental maladaptive behavior (i.e., delinquency), childhood exposure to parental violence/abuse/neglect, and deviant peer affiliations. For emotion recognition, boys with CD were 2.1 times more likely to have a deficit than girls with CD (*Wald* χ*^2^* = 4.19, *p* = *0.0*4), and a lower IQ among the youths with CD was also significantly associated with an increased likelihood of exhibiting a deficit in this domain (*Wald* χ*^2^* = 6.10, *p* = *0.0*13). For emotional learning, youths with CD whose mothers smoked during pregnancy were 3.2 times more likely to have a deficit in this domain than youths with CD whose mothers did not smoke during pregnancy (*Wald* χ*^2^* = 5.37, *p* = 0.021). None of the assessed risk factors predicted an emotion regulation deficit. Please note that none of the risk factors were associated with task performance in the TDCs.

## Discussion

The primary aim of this study was to investigate neuropsychological subgroups across three emotion processing domains linked to CD and test whether specific subgroups would map onto existing DSM-5 defined CD subtypes, including CD with vs. without the LPE specifier (4). Our dimensional analysis showed that, relative to TDCs, the CD group performed worse in all three emotion domains, but the largest case-control difference was found for the emotion recognition domain, followed by the emotion regulation domain, with the smallest difference observed for the emotion learning domain; this supports the findings of our previous report (17). Our categorical subgroup analysis substantiated this finding by revealing that deficient emotion recognition skills were the most common deficit in CD (~23%), followed by emotion regulation (~18%), with emotion learning the least common deficit (~13%). Critically, though, we also found that the majority of youths with CD (~56%) did not demonstrate meaningful deficits in any of the three emotion domains, and only a very small proportion of the CD sample showed pervasive deficits across all domains (i.e., deficits in all three emotion functions: ~1%). Overall, emotion processing deficits, if existent in youths with CD, appear to be unrelated to main phenotypic characteristics, such as age-of-onset of CD, symptom severity, or co-occurring psychiatric disorders. Moreover, contrary to our assumptions, the present data do not support notions that established clinical subtypes of CD, most importantly CD with vs. without the LPE specifier (1), are tightly linked to neurocognitive deficits in one particular emotion domain over another (i.e., emotion recognition deficits in CD+LPE vs. emotion regulation deficits in CD–LPE) (4, 6). Notably, being male and having a lower IQ increased the likelihood for showing a deficit in the emotion recognition domain, whereas maternal smoking during pregnancy increased the likelihood for having an emotion learning deficit. Please note, though, that our analytic approach to subdivide the CD sample into subgroups based on their neurocognitive performance (i.e., emotion domain deficit: “yes” or “no”) likely attenuated statistical power to detect associations with clinical correlates and associated risk factors.

Although the present findings support recent theoretical models emphasizing that CD is a remarkably heterogeneous condition, with different individuals being affected to different degrees in different domains of emotion functioning (4, 6, 7), our data also suggest that these influential neurocognitive models need to be modified to account for the substantial proportion of youths with CD who performed normally across all emotion processing domains studied here. Thus, future studies will need to (i) assess a broader range of emotion and non-emotion processes implicated in CD, such as executive function, decision-making, social cognition, and language skills (68, 69); (ii) investigate their performance profiles regarding neuropsychological heterogeneity; and then (iii) test their respective role in distinguishing between different developmental pathways of established and potentially novel clinical subtypes of CD, including testing the ability of each of these domains to predict the emergence or desistance of CD in high-risk groups as part of longitudinal studies (70).

It is, however, also conceivable that in fact emotion processing deficits only account for a proportion of youths with CD and that other biopsychosocial factors contribute to CD in those without such deficits (71). This idea is comparable to Moffitt's developmental taxonomy theory of CD suggesting that the adolescence-limited subtype does not suffer from neuropsychological deficits, whereas the early-onset and life-course-persistent subtype does (72) (see also (73) for a recent review). We note, however, that our data do not support this theory as age-of-onset of CD (although defined only retrospectively according to information from the K-SADS-PL interviews) was unrelated to the presence or absence of emotion processing deficits in this sample of youths with CD. This is consistent with previous smaller-scale studies showing no differences in emotion processing between CO-CD and AO-CD subtypes, including facial emotion recognition (74) and emotional learning (75), whereas both groups were deficient across these neuropsychological domains compared to TDCs. It should be stressed, though, that the empirical foundation of Moffitt's theory is not directly concerned with emotion functioning, but focuses instead on neurocognitive domains, such as reading, memory, vocabulary, and IQ (76). Therefore, more work is needed to determine the extent to which emotion functioning, in parallel to other neurocognitive mechanisms, contributes to different clinical manifestations and pathways within the CD population (including those with and without the LPE specifier) (77).

Both our dimensional and categorical data suggest that emotion recognition is the most consistently deficient neurocognitive domain in this sample of youths with CD. We can only speculate whether deficits in emotion recognition are more pivotal in the etiology of CD compared to both emotion learning and emotion regulation, or whether the specific task used to assess emotion recognition was simply more sensitive in detecting case-control differences than the two tasks that assessed the remaining emotion domains. Future studies might try to address this point, for instance, by creating tasks that are of comparable complexity and difficulty across various neurocognitive functions [e.g., emotion learning task (78), or emotion regulation task (79)].

Notably, our results neither confirm that youths with CD with the LPE specifier were disproportionately deficient in emotion recognition nor show that those without the LPE specifier displayed difficulties specifically in emotion regulation (4, 6). We did, however, find that being male and having a lower IQ—both well-documented risk factors for CD (50)—were associated with deficient emotion recognition skills [see for related findings (80, 81)], whereas maternal smoking during pregnancy—another well-known CD risk factor—increased the odds for deficient emotional learning; none of the risk factors tested here predicted an emotion regulation deficit. Although we are cautious in interpreting these results since we measured most of the risk factors retrospectively, the findings suggest that some biopsychosocial dispositions may increase the probability of developing specific types of emotion dysfunction, while others do not (or might do so only in interaction with other risk variables) (82). This idea is worth pursuing in future prospective longitudinal studies that examine a wider range of CD-related risk factors, including genetic and epigenetic processes (83–85), their complex interactions as well as their unique contribution to emotion dysfunctions as potential intermediate phenotypes at both the behavioral and brain level in CD.

This study had some additional limitations (see (17) for strengths and limitations regarding the sample composition): First, each emotion domain was assessed using only one experimental task which makes our neurocognitive battery less representative, thereby limiting the generalizability of the results. Follow-up studies should preferably apply more comprehensive test batteries including several tasks measuring overlapping emotion domains so as to replicate and extend our findings and, thus, obtain a richer understanding of emotion functioning in CD. Second, we stratified youths with CD as “deficient” or “intact” in terms of task performance, following a clinically motivated, person-centered subgrouping approach that is typical for neuropsychological assessments, highly relevant for clinical decision-making, and easily applicable by practitioners (86). However, the present procedure—and other “traditional” statistical data clustering methods aiming to fractionate clinical groups on the basis of neuropsychological scores—have been criticized for several reasons which go beyond the current study and cannot be discussed in depth here [but see (65)]. Notably, the most serious issue is that these clustering methods divide the data arbitrarily into a pre-specified number of severity classes regardless of the underlying data distribution (i.e., two classes here: “deficient” vs. “intact” individuals), and they always produce a result. This sometimes leads to even unmanageably small subgroups, such as the 1% of youths with CD who were found to show deficits across all three emotion domains in the present study. Thus, alternatively, more elaborate machine-learning approaches, such as “normative modeling” (87), have been proposed to map neuropsychological variation with the advantage of not making strong *a priori* assumptions about the existence or number of subgroups with abnormal task performance (i.e., defined as extreme value, or “outlier,” from the normative range). It will be interesting to see how these novel subgrouping algorithms complement traditional clustering methods in identifying distinct neurocognitive subtypes of CD with potentially unique clinical profiles and underlying biology.

In conclusion, the current findings provide first evidence that youths with CD display strikingly diverse profiles in neuropsychological performance across three domains of emotion processing that have previously been linked to CD etiology, including emotion recognition, emotion learning, and emotion regulation (4). Similar to findings in ADHD (88), we were able to reveal different neurocognitive subgroups in which emotion functioning was deficient to varying degrees, with a sizable subgroup of CD cases showing no meaningful emotion processing deficits at all. Clearly, deficits within a specific emotion processing domain may be clinically important for only a subgroup of patients, but not for the entire CD population. Consequently, treatments targeting emotion processing may be beneficial for some, but not all, individuals with CD (12, 89, 90). Whether, and if so which, neuropsychological interventions in non-emotion domains may be required to help patients with CD who have intact emotion processing skills needs to be evaluated in future studies. Importantly, while the current study should be regarded as exploratory and illustrative since we used, for the first time, a common-metric analytic approach from pediatric neuropsychology in order to subgroup youths with CD based on their emotion processing performance, novel classification algorithms based on machine learning are warranted to assist in identifying and validating distinct and meaningful neurocognitive phenotypes of CD, ideally replicating and thus substantiating the findings of this study.

## Data Availability Statement

The datasets generated for this study are available on reasonable request in line with the data sharing policy of the FemNAT-CD consortium.

## Ethics Statement

Written informed consent was obtained for all participants after they were informed about the experimental procedures and the aims of the study. Local ethics committees at all sites of the FemNAT-CD consortium reviewed and approved the study protocol. The study was carried out in accordance with the recommendations of good clinical practice and in accordance with the Declaration of Helsinki and national legislation.

## Author Contributions

GK, GF, and KK conceived the current study. AB, AM, AS, KG-M, AW, JR, RP, HO, LJ, AR, LK, JA, and SB recruited participants and carried out assessments and data collection. GF, BH-D, AV, HL, AH, AF-R, AP, CS, SD, CF, and KK supervised the study. GK performed the statistical analyses and drafted the manuscript. GF, CS, SD, CF, and KK contributed to writing parts of the manuscript. CF coordinated the project which this study was part of. All authors read, commented on drafts of the paper, and approved the final manuscript and its submission.

## Conflict of Interest

KK has received speaker fees from Shire Pharmaceuticals and Medice. CS receives royalties for a book on aggression. CF receives royalties for books on ADHD, ASD, and Depression. SD has received speaker fees from the Child Mental Health Centre and the Centre for Integrated Molecular Brain Imaging. The remaining authors declare that the research was conducted in the absence of any commercial or financial relationships that could be construed as a potential conflict of interest.
