# Supplementary material for: Neuropsychological Subgroups of Emotion Processing in Youths With Conduct Disorder
Source: Front Psychiatry. 2020 Dec 22;11:585052. doi: 10.3389/fpsyt.2020.585052 (PMC7783416; doi:10.3389/fpsyt.2020.585052)
Supplement: Supplementary file 1 [file Data_Sheet_1.docx]

Supplementary material for “Neuropsychological Subgroups of Emotion Processing in Youths with Conduct Disorder” by Kohls et al.

# Materials and Methods

***Participants***

**Table S1** Number of participants per group by clinical site

| **Site** | **Total**  *N* | **TDC**  *n* | **CD**  *n* | *χ^2^(df=9)* |
| --- | --- | --- | --- | --- |
| Frankfurt | 209 | 124 | 85 | 22.6 (*p*=.007) |
| Aachen | 268 | 140 | 128 |  |
| Amsterdam | 125 | 80 | 45 |  |
| Southampton | 146 | 89 | 57 |  |
| Basel | 53 | 30 | 23 |  |
| Birmingham | 162 | 105 | 57 |  |
| Barcelona | 33 | 13 | 20 |  |
| Bilbao | 109 | 56 | 53 |  |
| Szeged | 38 | 14 | 24 |  |
| Athens | 109 | 59 | 50 |  |
|  | **1252** | **710** | **542** |  |

*Note: CD = conduct disorder; TDC = typically developing controls.*

Please note that we excluded 13.5% of participants with incomplete neuropsychological data, comprising 17% of youths with CD and 7% of TDCs. The main reasons for missing data were (1.) technical difficulties (e.g., software problems), (2.) experimenter errors (e.g., no data logging), (3.) lack of time, (4.) noncompliance by the participant, or (5.) study dropout. We assume that the incomplete neuropsychological data are missing at random due to the various reasons listed above. Importantly, when comparing the excluded cases with the included cases, we did not find significant differences in age, IQ, comorbidities, or CD severity (all *p*s >.05), suggesting that the reported data for CD vs. TDCs are not biased by excluding participants with incomplete data sets.

# Results

**Table S2** Bivariate correlations between main performance variables and age by group

|  | **Emotion regulation** | **Emotion recognition** | **Emotion learning** |
| --- | --- | --- | --- |
| TDC (n=710) | *r* = -.53 | *r* = -.39 | *r* = -.22 |
| CD (n=542) | *r* = -.43 | *r* = -.25 | *r* = -.08 |
| **TDC vs. CD: Fisher’s *z (p)*** | **.011** | **.003** | **.006** |

*Note: CD = conduct disorder; TDC = typically developing controls.*
